# Functional salivary gland regeneration by transplantation of a bioengineered organ germ

**DOI:** 10.1038/ncomms3498

**Published:** 2013-10-01

**Authors:** Miho Ogawa, Masamitsu Oshima, Aya Imamura, Yurie Sekine, Kentaro Ishida, Kentaro Yamashita, Kei Nakajima, Masatoshi Hirayama, Tetsuhiko Tachikawa, Takashi Tsuji

**Affiliations:** 1 Research Institute for Science and Technology, Tokyo University of Science, Noda 278-8510, Japan; 2 Division of Research and Development, Organ Technologies Inc, Tokyo 01-0048, Japan; 3 Department of Biological Science and Technology, Graduate School of Industrial Science and Technology, Tokyo University of Science, Noda 278-8510, Japan; 4 Department of Clinical Pathophysiology, Tokyo Dental College, Chiba 261-8502, Japan; 5 Department of Ophthalmology, Keio University School of Medicine, Tokyo 160-8582, Japan; 6 Department of Oral Pathology, Showa University School of Dentistry, Tokyo 145-8515, Japan

## Abstract

Salivary gland hypofunction, also known as xerostomia, occurs as a result of radiation therapy for head cancer, Sjögren’s syndrome or aging, and can cause a variety of critical oral health issues, including dental decay, bacterial infection, mastication dysfunction, swallowing dysfunction and reduced quality of life. Here we demonstrate the full functional regeneration of a salivary gland that reproduces the morphogenesis induced by reciprocal epithelial and mesenchymal interactions through the orthotopic transplantation of a bioengineered salivary gland germ as a regenerative organ replacement therapy. The bioengineered germ develops into a mature gland through acinar formations with a myoepithelium and innervation. The bioengineered submandibular gland produces saliva in response to the administration of pilocarpine and gustatory stimulation by citrate, protects against oral bacterial infection and restores normal swallowing in a salivary gland-defective mouse model. This study thus provides a proof-of-concept for bioengineered salivary gland regeneration as a potential treatment of xerostomia.

## Abstract (web summary)

Salivary gland dysfunction as a result of diseases or ageing reduces the quality of life and causes various oral health problems. Here the authors show that the salivary gland function of mice can be recovered by orthotopic transplantation of a bioengineered salivary gland germ.

## Graphical abstract

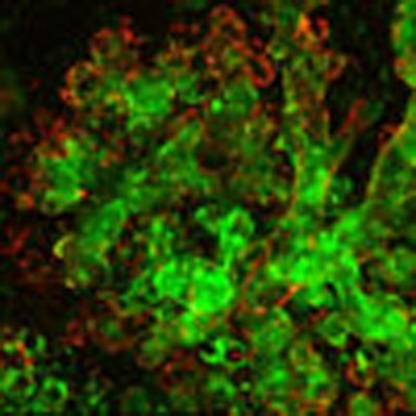

Salivary glands have essential roles in normal upper gastrointestinal tract function and oral health, including the digestion of starch by the salivary amylase, swallowing and the maintenance of tooth hard tissues through the production of saliva^1^,^2^. There are three major salivary glands—the parotid, submandibular and sublingual glands—as well as minor salivary glands^3^,^4^,^5^,^6^,^7^. Salivary glands arise from their organ germ, which is induced by reciprocal epithelial and mesenchymal interactions during embryogenesis^6^. The oral epithelium forms bud along with the underlying mesenchyme at embryonic day 12.5 (ED12.5), followed by an early network of epithelial branches with terminal buds (ED14.5), and then interacts with the lumen, which develops from a majority of ducts between the canalicular (ED15.5) and terminal bud stage (ED18.5)^3^. Stem cells in the terminal bulb portions undergo cellular proliferation and subsequent differentiation into acinar and duct cells as well as myoepithelial cells, and develop into the duct system in coordination with acinar cytodifferentiation^5^. Terminal bulb cells differentiate into intercalated duct cells and are stored as stem cells to develop into acinar, myoepithelial and duct cells^7^. Saliva secreted by an acinar cell in response to nerve stimulation contributes to oral health and quality of life^6^.

The salivary gland impairment, which results from various physiological conditions such as radiation therapy for head and neck cancer, Sjögren’s syndrome and aging^8^, leads to acinar cell damage and salivary gland hypofunction, including xerostomia (dry mouth syndrome)^9^,^10^. Xerostomia is known to induce various clinical problems in oral health, including dental decay, bacterial infection, mastication dysfunction, swallowing dysfunction, dysgeusia and a general reduction in quality of life^8^. Current therapies for xerostomia involve the administration of artificial saliva substitutes and sialogogues, which are drugs or substances that increase the flow rate of saliva^11^,^12^. In addition, parasympathomimetic drugs such as pilocarpine and cevimeline, which act on the muscarinic M3 receptor to induce salivary flow, have been used to stimulate residual functional salivary gland tissues^12^,^13^. However, these saliva substitutes and drugs cannot restore salivary gland function^11^,^14^. Therefore, a novel therapeutic treatment for the restoration of salivary gland function is needed^14^.

One concept in regenerative therapy involves tissue stem cell transplantation to restore damaged tissues and organs in a variety of diseases^15^,^16^,^17^. In salivary gland regeneration, the transplantation of various stem cells, such as intercalated duct cells connecting terminal acini and striated ducts of the salivary gland^18^, c-kit-positive duct cells in the excretory duct of human salivary glands^19^, salivary gland-derived progenitor cells isolated from duct-ligated animals and bone marrow-derived Sca-1- and c-kit-positive cells, has been reported for salivary gland tissue repair^20^,^21^. Further, organ replacement regenerative therapy, which can replace lost or damaged organs following disease, injury or aging with a fully functional bioengineered organ, is also expected to provide a novel therapeutic strategy for organ transplantation^22^,^23^,^24^. Our recent studies have provided proofs-of-concept that fully functional regeneration of ectodermal organs such as teeth and hair follicles can be achieved by the transplantation of bioengineered organ germs that were reconstituted by our organ germ method^25^, in organ replacement regenerative therapy^25^,^26^,^27^.

Here we demonstrate the regeneration of fully functional salivary glands through the orthotopic transplantation of a bioengineered salivary gland germ in adult mice. The serous- and mucous-acinar cell types, as well as intercalated and striated ducts of the bioengineered salivary gland, depended on the origin of the epithelial and mesenchymal cells isolated from the submandibular or sublingual gland germ. The bioengineered submandibular gland, which was transplanted using an interepithelial tissue-connecting plastic method, produced saliva in response to the administration of pilocarpine and gustatory stimulation by citrate, protected against oral bacterial infection and restored swallowing in a mouse model of a salivary gland defect. The current study illustrates the potential for bioengineered salivary gland regeneration.

## Results

### Generation of a bioengineered salivary gland germ

**Figure 1:**
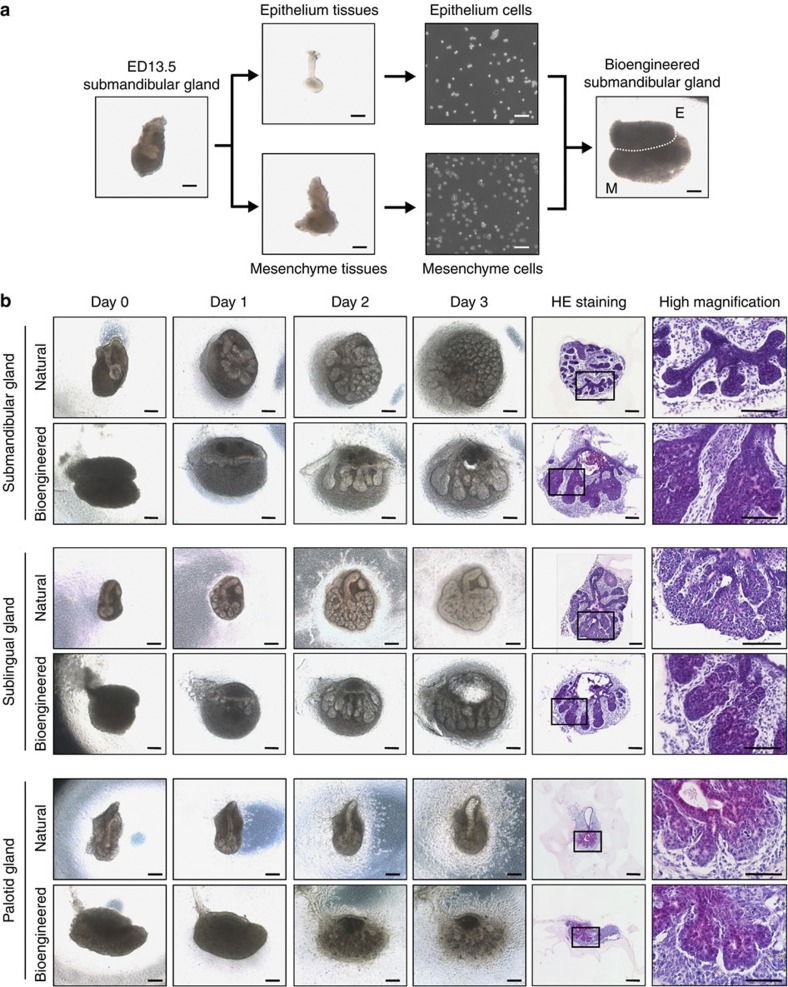
Generation of a bioengineered salivary gland germ. (**a**) Phase-contrast images of the ED13.5 submandibular gland germ, tissues, dissociated single cells and the bioengineered submandibular gland germ, which was reconstituted using the organ germ method. E, epithelial cells; M, mesenchymal cells. Scale bar, 200 μm. (**b**) Phase-contrast images of natural (upper) and bioengineered (lower) salivary gland germs, including the ED13.5 submandibular gland (top columns), the ED14.5 sublingual gland (middle columns) and the ED14.5 parotid gland (bottom columns) on days 0, 1, 2 and 3 of organ culture. The salivary glands were analysed by haematoxylin and eosin staining on day 3 of organ culture. Scale bar, 200 μm.

We first investigated whether each bioengineered salivary gland germ, including the parotid, submandibular and sublingual glands, has the ability to regenerate into mature glands using our previously developed organ germ method (Fig. 1a)^25^. The bioengineered gland germs were reconstituted with epithelial and mesenchymal single cells isolated from each gland germ at ED13.5–14.5 (Fig. 1a). After 1 day in organ culture, the bioengineered salivary gland germs exhibited epithelial–mesenchymal interactions and had developed to an initial bud stage (Fig. 1b). After 3 days in organ culture, the bioengineered salivary gland germs, including the parotid, submandibular and sublingual gland germs, underwent branching morphogenesis followed by stalk elongation and cleft formation (Fig. 1b). From 3 days of organ culture onwards, the accumulation of saliva could be observed in the ducts of the bioengineered gland germs (Fig. 1b).

### Engraftment of a bioengineered salivary gland

**Figure 2:**
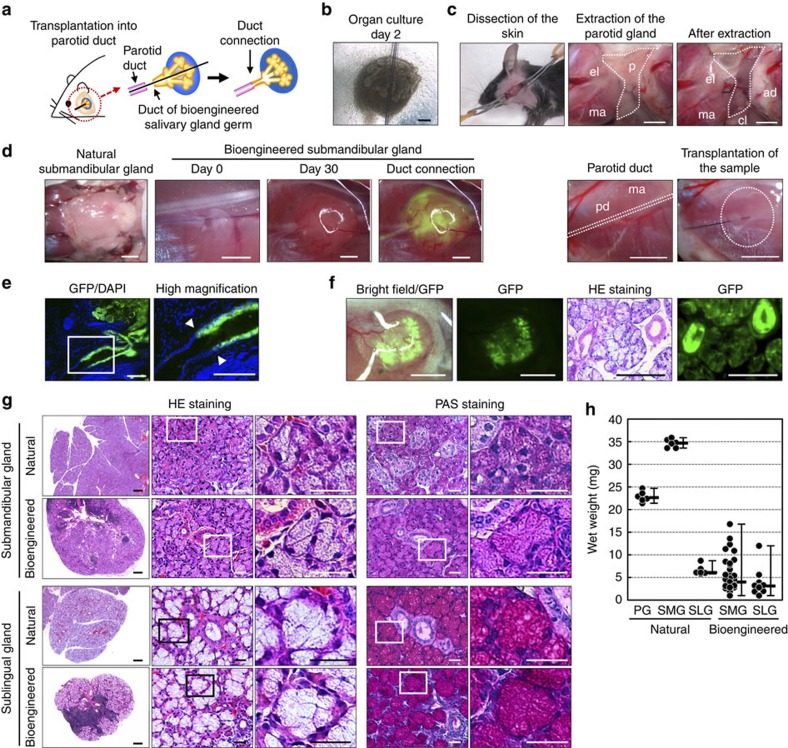
Transplantation of a bioengineered salivary gland. (**a**) Schematic representation of the transplantation procedure using the interepithelial tissue-connecting plastic method with the bioengineered salivary gland germ. (**b**) Phase-contrast images of the bioengineered salivary gland germ containing a PGA monofilament guide. Scale bar, 200 μm. (**c**) Photographs of bioengineered salivary gland germ transplantations in salivary gland defect mice. The three major salivary glands were extracted and a bioengineered salivary gland germ was transplanted. Scale bar, 1 mm. (**d**) Photographs of the natural submandibular gland (left) and the bioengineered salivary gland at days 0 and 30 after transplantation (second and third figure from the left). FITC-gelatine conjugate was injected into the bioengineered submandibular gland from the host parotid duct (right). Scale bar, 1 mm. (**e**) Histological images of the duct connection between the host duct and epithelial duct of the GFP-labelled bioengineered salivary gland (left). Higher magnification images in the box area are shown (right). Bioengineered salivary glands developed *in vivo* with the correct connection to the recipient parotid gland duct (arrowhead). Scale bar, 150 μm. (**f**) Photographs of the bioengineered salivary gland, which was reconstituted from GFP-transgenic mice-derived epithelial cells and normal mice-derived mesenchymal cells (left: merged with the stereomicroscope image and the GFP image, second figure from the left: GFP image). Scale bar, 1 mm. The section images of haematoxylin and eosin (HE) staining (third figure from the left) and GFP fluorescence (right) are shown. Scale bar, 200 μm. (**g**) Histological analysis of the submandibular gland (upper columns) and the sublingual gland (lower columns), including the natural (upper) and bioengineered (lower) salivary glands. Images of HE staining (left three) and periodic acid and Schiff (PAS) staining (right two) are shown. Higher magnification images in each box area are shown (second and third panels from the left, right figure). Scale bar, 100 μm in the left column and 25 μm in the second and subsequent columns. (**h**) Wet weights of natural and bioengineered salivary glands. The data are presented as the median±max, min; *n*=6 for the natural parotid, submandibular and sublingual glands, *n*=20 for the bioengineered submandibular glands and *n*=9 for the bioengineered sublingual glands. PG, parotid gland; SLG, sublingual gland; SMG, submandibular gland.

We next investigated whether a bioengineered salivary gland germ could be engrafted and developed by integrating between the host salivary duct and the bioengineered salivary duct in a murine model of a salivary gland defect. The model was prepared by extracting the parotid gland, submandibular gland and sublingual gland from 7-week-old mice. The bioengineered salivary gland germ, including submandibular and sublingual gland germs, was transplanted using our previously developed interepithelial tissue-connecting plastic method with a guide for duct direction inserted into the bioengineered germ (Fig. 2a–c)^27^. Bioengineered salivary glands developed *in vivo* with the correct connection to the recipient parotid gland duct, which was confirmed by the transport of a fluorescence substance injected into the host parotid duct to the transplanted bioengineered salivary gland without leaking (Fig. 2d). In a bioengineered submandibular gland germ that was reconstituted from green fluorescence protein (GFP) mice-derived epithelial cells and normal mice-derived mesenchymal cells, the correct duct connection, the GFP-positive duct of the bioengineered salivary gland and the non-fluorescence parotid duct in the recipient mice were confirmed (Fig. 2e). Green fluorescence was also detected in the acinar duct, including acinar cells and the myoepithelium (Fig. 2f). Histological analysis using haematoxylin and eosin staining and periodic acid and Schiff staining revealed a distinctive acinar structure, including serous acinar cells, in the bioengineered submandibular gland and mucous acinar cells in the bioengineered sublingual gland as well as in the natural salivary glands (Fig. 2g). The wet weights of the bioengineered submandibular (median: 4.0 mg, minimum (min): 1.0 mg, maximum (max): 16.8 mg) and sublingual glands (median: 3.1 mg, min: 1.0 mg, max: 12.0 mg) were relatively low compared with the weights of the natural submandibular (median: 34.7 mg, min: 33.6 mg, max: 35.9 mg) and sublingual glands (median: 6.1 mg, min: 5.8 mg, max: 8.7 mg; Fig. 2h). These results indicate that the bioengineered salivary gland developed with properties corresponding to the origins of the stem cells isolated from embryonic organ germs.

### Functional analysis of bioengineered saliva secretion

**Figure 3:**
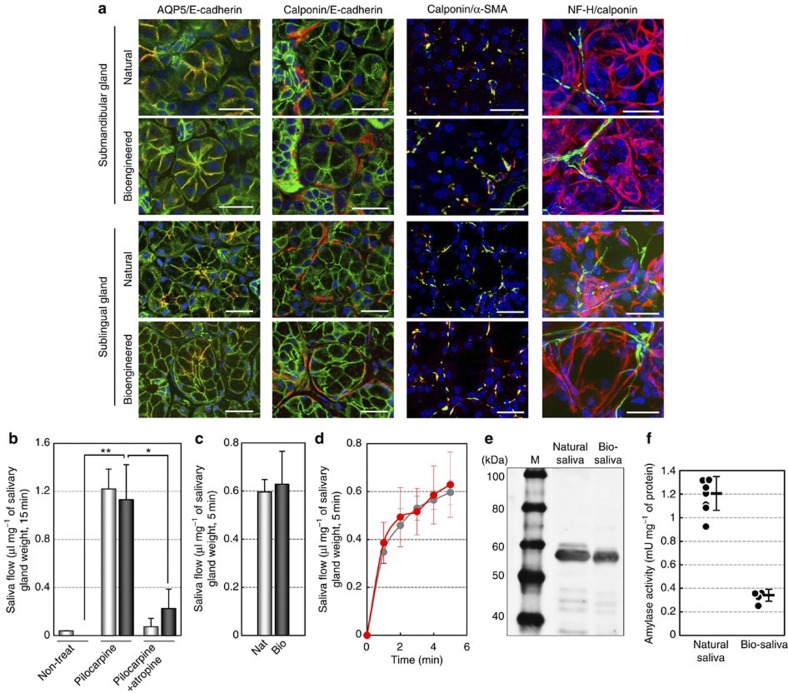
Assessment of saliva secretion after the salivary gland regeneration. (**a**) Immunohistochemical analysis of the submandibular gland and the sublingual gland, including the natural (upper) and bioengineered (lower) salivary glands. Merged images of AQP5 (red) and E-cadherin (green; left columns), calponin (red) and E-cadherin (green; second columns from the left), calponin (red) and α-smooth muscle cell actin (green; third columns from the left) and calponin (red) and NF-H (green; right columns) are shown. Scale bar, 25 μm. (**b**) Assessment of the amount of saliva secretion associated with normal mice (light bars) and bioengineered submandibular gland-engrafted mice (dark bars) before and after the administration of pilocarpine, without or with atropine. The data are shown as the mean±s.e.m.; *n*=10 for normal and *n*=7 for the bioengineered submandibular gland. *P<0.05, **P<0.001 by Student’s *t*-test. (**c**) Assessment of the amount of saliva secretion associated with normal mice (light bars) and bioengineered submandibular gland-engrafted mice (dark bars) after gustatory stimulation by citrate. Nat, normal mice (light bar); Bio, bioengineered submandibular gland-engrafted mice (dark bar). The data are shown as the mean±s.e.m.; *n*=5 for normal and bioengineered submandibular glands. (**d**) The time course of the amount of saliva secretion associated with normal mice (grey dot) and bioengineered submandibular gland-engrafted mice (red dot) every 1 min for 5 min after gustatory stimulation by citrate. The data are shown as the mean±s.e.m.; *n*=5 for normal and bioengineered submandibular glands. (**e**) Western blot analysis of amylase protein in saliva secreted from natural (center) and bioengineered submandibular glands (right). M, size marker. (**f**) Analysis of the amylase activities of saliva secreted from natural and bioengineered submandibular glands. The data are shown as the mean±s.e.m.; *n*=7 for natural saliva and *n*=4 for bioengineered submandibular gland saliva.

Saliva secretion is an essential role of salivary glands for maintaining oral and general homoeostasis and should be restored by salivary gland regeneration. We next analysed the acinar cell differentiation and acinar formation of engrafted bioengineered salivary glands. In bioengineered submandibular and sublingual glands, the localization of the membrane water channel protein aquaporin-5 (AQP5) was observed at the apical membrane of acinar cells, as it is in natural salivary glands (Fig. 3a). E-cadherin and calponin proteins were detected in acinar and duct cells as well as in myoepithelial cells, which enveloped acinar cells in these bioengineered glands (Fig. 3a). The expression of α-smooth muscle cell actin protein was also detected in calponin-positive myoepithelial cells in these bioengineered glands (Fig. 3a). Innervations were also detected in the interstitial tissue among acinar cells, and neurofilament H (NF-H)-expressing nerve fibres connected to calponin-positive myoepithelial cells (Fig. 3a). These results indicate that engrafted bioengineered salivary gland germ cells successfully formed correct tissue structures and are potentially able to secrete saliva in response to neural stimulation.

We therefore investigated whether bioengineered salivary glands are capable of secreting saliva in response to nerve stimulation. Salivary secretion after the administration of pilocarpine, which is a parasympathetic nerve-irritating agent, was significantly induced in bioengineered submandibular gland-engrafted mice compared with salivary gland defect mice (Fig. 3b). No significant difference in saliva flow between the natural mice and the bioengineered submandibular gland-engrafted mice was observed. By contrast, saliva secretion in natural and bioengineered submandibular gland-engrafted mice was inhibited by the anticholinergic agent atropine (Fig. 3b). We further analysed biological salivary secretion using gustatory stimulation with citrate^28^. The engrafted bioengineered salivary glands could secrete significant quantities of saliva in response to citrate stimulation via afferent and efferent neural networks. These results indicate that the bioengineered salivary gland secreted saliva via the proper nerve innervations and neurotransmission under the control of the central nervous system (Fig. 3c,d).

Next, we analysed the protein components, such as amylase, secreted from the bioengineered submandibular gland in saliva. Amylase was detected in the bioengineered saliva using western blot analysis (Fig. 3e). The mean±s.e.m of the amylase activity of the bioengineered saliva was relatively low (0.342±0.05 mU mg^−1^) compared with the level in natural saliva (1.206±0.14 mU mg^−1^; Fig. 3f). These results indicate that the functional ability of a bioengineered salivary gland to secrete saliva containing amylase activity in response to cholinergic stimulation is comparable to that of a natural salivary gland.

### Amelioration of dry mouth symptoms by saliva secretion

**Figure 4:**
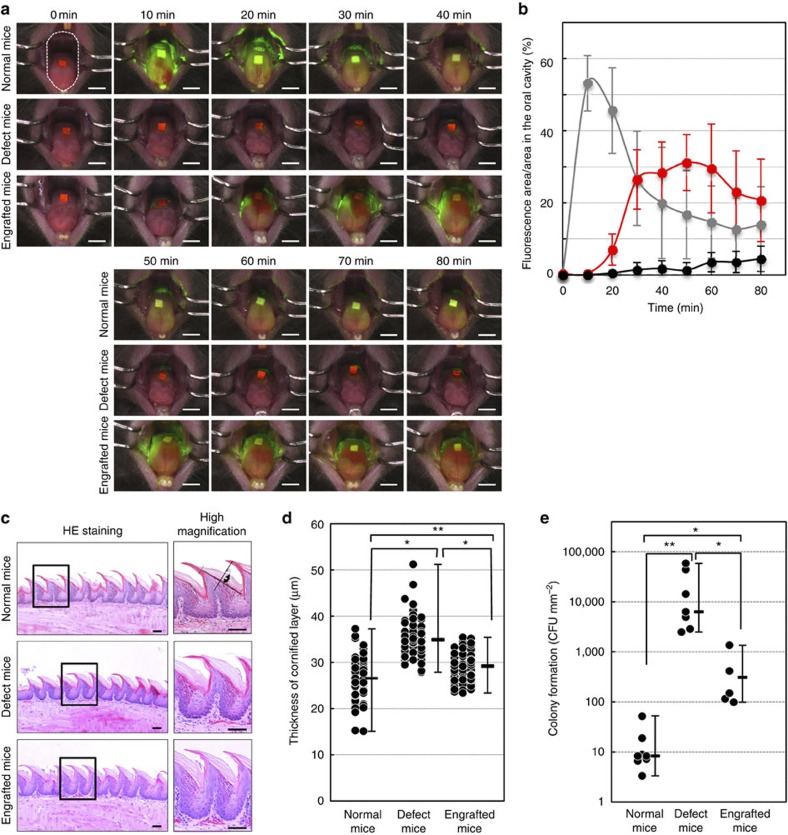
Improvement of xerostomia by the salivary gland regeneration. (**a**) Time course images of diffusion and the washing effect of secreted saliva acquired every 10 min for 60 min after pilocarpine stimulation of normal mice (top columns), salivary gland defect mice (middle columns) and bioengineered submandibular gland-engrafted mice (bottom columns). The measurement area of fluorescein diffusion in the oral cavity are shown (top columns and left). Scale bar, 2 mm. (**b**) Assessment of the diffusion and cleansing effect of secreted saliva in normal mice (grey dots), salivary gland defect mice (black dots) and bioengineered submandibular gland-engrafted mice (red dots). The data are presented as the mean±s.e.m; *n*=5 for normal mice, *n*=5 for salivary gland defect mice and *n*=5 for bioengineered submandibular gland-engrafted mice. (**c**) Histological analysis of the lingual epithelium in filiform papillae (left) and a higher magnification image (right) of normal mice, salivary gland defect mice and the bioengineered submandibular gland-engrafted mice. The measurement width of epithelial hyperplasia in the cornified layer of filiform papillae are shown (top columns and right). Scale bar, 50 μm. (**d**) Assessment of hyperplasia in the cornified layer of filiform papillae in normal mice, salivary gland defect mice and bioengineered submandibular gland-engrafted mice. The data are shown as the median±max, min; *n*=60 for filiform papillae in normal mice, *n*=90 for filiform papillae in salivary gland defect mice and *n*=90 for filiform papillae in bioengineered submandibular gland-engrafted mice. *P<0.003, **P<0.001 by Student’s *t*-test. (**e**) Assessment of bacterial propagation in the buccal mucosa of normal mice, salivary gland defect mice and bioengineered submandibular gland-engrafted mice. The data are presented as the median±max, min; *n*=9 for normal mice, *n*=7 for salivary gland defect mice and *n*=5 for bioengineered submandibular gland-engrafted mice. *P<0.05, **P<0.001 by Student’s *t*-test.

Saliva secretion is essential to the maintenance of oral health, and xerostomia causes various health problems, including dental caries, periodontal disease, bacterial infection and swallowing dysfunction^8^,^11^. We next investigated whether the transplantation of a bioengineered salivary gland could ameliorate dry mouth symptoms by cleansing the oral cavity and inhibiting oral bacterial growth. To semiquantitatively analyse saliva secretion, we performed a fluorescein diffusion analysis. Briefly, fluorescent sodium test paper, which contains a fluorescent dye dissolved in saliva drippings, was placed on the tongue. Next, pilocarpine was administered and the amount of fluorescence in the oral cavity was measured. In control natural mice, the amount of fluorescence increased at 10 min and then gradually decreased within 60 min (Fig. 4a,b). By contrast, dye diffusion was not observed during the observation period in salivary gland defect mice (Fig. 4a,b). Gradual diffusion and disappearance of the fluorescein dye in the oral cavity was observed in mice transplanted with the bioengineered submandibular gland (Fig. 4a,b). Xerostomia induces hyperplasia in the cornified layer of filiform papillae^29^. The thickness of the cornified layer of filiform papillae in salivary gland defect mice (median: 34.9 μm, min: 27.9 μm, max: 51.2 μm) was significantly greater than the thickness in normal mice (median: 26.5 μm, min: 15.1 μm, max: 37.27 μm, Fig. 4c,d). In mice that were engrafted with a bioengineered submandibular gland, the thickness of the cornified layer was significantly reduced (median: 29.2 μm, min: 23.3 μm, max: 35.4 μm) compared with the defect mice (Fig. 4c,d). Another function of saliva is its antibacterial activity, which has an important role in the maintenance of homoeostasis in the oral cavity^11^. The number of oral bacteria was drastically increased in salivary gland defect mice compared with normal mice (Fig. 4e). The number of bacterial colonies that formed in the mice engrafted with the bioengineered submandibular gland germ was significantly reduced compared with the number in salivary gland defect mice (Fig. 4e). These results indicate that the bioengineered submandibular gland has a cleansing function, which prevents dryness and inhibits bacterial growth by secreting saliva into the oral cavity.

### Functional recovery of swallowing and survival

**Figure 5:**
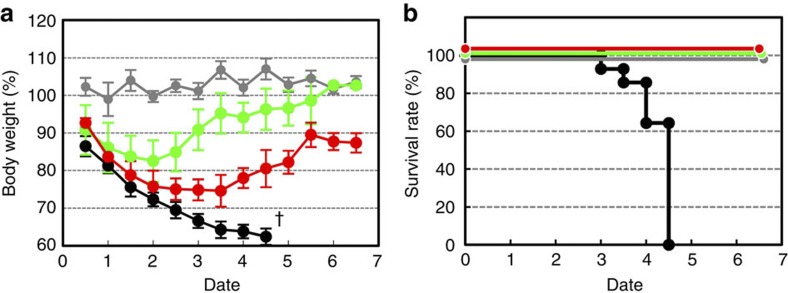
Analysis of body weight fluctuation and survival rate. (**a**) Measurement of body weight every 0.5 days after transplantation in normal mice (grey dots, *n*=6), salivary gland defect mice (black dots, *n*=11), salivary gland-engrafted mice (red dots, *n*=5) and salivary gland defect mice were given high-viscosity water (green dots, *n*=5). All salivary gland defect mice died at 4 days (†) after the removal of the salivary glands, including the parotid, submandibular and sublingual glands. (**b**) Survival rates of salivary gland defect mice (*n*=11) and bioengineered submandibular gland-engrafted mice (*n*=5).

Among salivary gland functions, the swallowing function is critical for nutrition and reducing the risk of aspiration, which can cause chronic lung disease as well as affecting survival and quality of life, including health and aging^8^,^11^. To investigate the restoration of the swallowing function by the transplantation of the bioengineered salivary gland germ, we analysed the body weight and survival of salivary gland defect mice (Fig. 5). After the extraction of the salivary glands from the model mice, their body weight abnormally decreased (Fig. 5a) and all died within 5 days of the surgery (Fig. 5b), although the mice were supplied with sufficient food and water. The feeding of high-viscosity water at ~1 Pa·s prevented the decrease in body weight and survival rate of salivary gland defect mice. Thus, the symptoms observed after the extraction of the salivary glands may be because of swallowing dysfunction. Body weight initially decreased within 2 days in mice transplanted with bioengineered submandibular gland germs; however, it recovered and increased 4 days after transplantation, which is the approximate time needed for the engrafted germ to develop into acini *in vivo* (Fig. 5a). All of the transplanted mice survived, and the rate dramatically improved (Fig. 5b). These results indicate that saliva secretion from bioengineered salivary glands has an essential role in the swallowing function associated with the maintenance of oral health as well as general health.

## Discussion

Our current study demonstrates that bioengineered submandibular glands form a fully functional salivary gland that produces a sufficient volume of saliva in response to pilocarpine and taste stimulation for maintenance of oral conditions and for recovery of swallowing. These findings provide a proof-of-concept for bioengineered salivary gland regeneration as a potential treatment of xerostomia.

Saliva has an essential role in oral health, and the reduction of saliva flow causes a deterioration in quality of life. The salivary gland regeneration by various stem cell transplantation strategies has been proposed and attempted in several previous studies to repair salivary gland impairment^3^,^14^. Previous studies have demonstrated the potential for acini and duct formation on the transplantation of various epithelial stem cells, such as intercalated duct cells that connect the terminal acini and striated duct of salivary glands^20^,^30^ and c-kit-positive duct cells in the excretory duct of human salivary glands^19^. Bone marrow-derived Sca-1- and c-kit-positive cells have been reported to rescue salivary gland function through the recovery of saliva production^21^. We have also proposed a concept for bioengineered tooth or hair follicle organ replacement, but not tissue repair, via the transplantation of their germs as a next generation of regenerative therapy^26^,^27^. In the current study, we demonstrate the functional replacement of the salivary gland by orthotopic transplantation of the bioengineered germ in a mouse model of the salivary gland defect^26^. Thus, this study provides a proof-of-concept for the bioengineered salivary gland replacement as a way to regenerate secretory organs. Bioengineered salivary gland replacement would be a useful therapeutic strategy for severe salivary gland impairment caused by irradiation, Sjögren’s syndrome or aging, although the identification of the cell sources for obtaining a bioengineered germ from a patient remains a critical issue.

Saliva, which is produced from serous- and mucous-type acinar cells such as serous-, seromucous- and mucous-type acinar cells in the murine parotid, submandibular and sublingual glands, respectively, has essential roles in oral function^3^,^6^. The dominant acinar cell type in these salivary glands changes and differentiates during the salivary gland morphogenesis^3^,^6^. Previously, recombination experiments between epithelium and mesenchyme isolated from germs of the mammary gland and the pouch of Rathke demonstrated that organ fate is dependent on the epithelium of organ germs in secretory organs^24^. Different gene expression profiles were also observed in the epithelium of developing submandibular and sublingual glands and contributed to the determination of the dominant cell type as well as the cytodifferentiation of acinar cells^7^. In the current study, we demonstrated that the distinction between the serous- and mucous-type acinar cells of bioengineered salivary glands was dependent on the origin of the epithelial and mesenchymal stem cell sources, which were isolated from salivary gland germs for the reconstitution of the bioengineered germ. The distinction may also be regulated by the combination of germ cells isolated from the submandibular and sublingual glands. These results indicate the potential to properly regenerate the appropriate serous/mucous distinction of the bioengineered salivary gland via the cell processing of the bioengineered salivary gland germ.

The peripheral parasympathetic and sympathetic nervous systems have important roles in the secretion of saliva via the various striated ducts entering the oral cavity^31^, and in the salivary gland development and regeneration^32^. Salivary secretion as an appropriate biological response is evoked by various stimuli such as mastication and taste through the gustatory receptors, mechanoreceptors, nociceptors and olfactory receptors^31^. Acetylcholine, a component of the parasympathetic nervous system, regulates water production through AQP5, which is activated by the M3 receptor on acinar cell membranes and the secretion of saliva from acini^33^. By contrast, the secretion of protein components in saliva, which are stored in secretory granules located at the side of the duct, are known to be regulated by the activation of sympathetic nerves^31^. The restoration of these nervous systems is critical and must be addressed by the salivary gland replacement regenerative therapy as well as in the replacement of other organs^34^,^35^. Recently, we demonstrated the successful restoration of nervous systems in tooth and hair follicle regeneration, which achieved the proper perceptive potential of noxious stimulation in tooth tissues^26^ and piloerection using surrounding arrector pili muscles through the activation of sympathetic nerves^27^, respectively. In the current study, the innervation of the bioengineered submandibular gland and the inductive potential of saliva secretion through gustatory stimulation by citrate were observed. These results suggest the functional restoration of nervous systems, including not only parasympathetic and sympathetic nerves but also afferent nerves, for saliva secretion. The process and contribution of the innervation of these nerves during the salivary gland organ regeneration remains to be elucidated.

In conclusion, the current study provides novel evidence of successful replacement of a fully functional salivary gland through the transplantation of a bioengineered germ. Our findings indicate that bioengineered organ replacement, which can achieve the fully functional restoration of organ function, is applicable to a wide variety of ectodermal placode organs. Further studies on the identification and characterization of adult tissue stem cells for the salivary gland replacement will facilitate the future development of the salivary gland regeneration. Further investigation of the methods for the clinical application including engraftment and recipient’s niches for organ regeneration will contribute to the development of the salivary gland regeneration therapy in human.

## Methods

### Animals

C57BL/6 mice were purchased from SLC Inc. (Shizuoka, Japan). C57BL/6-TgN (act-EGFP) OsbC14-Y01-FM131 mice were obtained from Japan SLC and the RIKEN Bioresource Center (Tsukuba, Japan), respectively. Mouse care and animal handling were performed in accordance with the National Institutes of Health guidelines. All experimental protocols were approved by the Animal Care and Use Committee of Tokyo University of Science.

### Reconstitution of bioengineered salivary gland germs

Submandibular glands were dissected from the mandibles both of male and female of ED13.5 or ED14.5 mice. Sublingual glands and parotid glands were obtained from ED14.5 mice. Isolated salivary glands were treated with 50 U ml^−1^ dispase (BD, Franklin Lakes, NJ, USA) for 1.5 min at room temperature and then separated into epithelial and mesenchymal tissues. Epithelial tissues were treated twice at 37 °C for 10 min with 100 U ml^−1^ collagenase I (Worthington, Lakewood, NJ, USA) in Ca^2+^- and Mg^2+^-PBS(−). Next, the tissues were treated with PBS(−) supplemented with 0.25% trypsin (Sigma-Aldrich Japan, Tokyo, Japan) for 5 min at 37 °C and dissociated into single cells by gentle pipetting. Cells of mesenchymal origin were also prepared by treatment with PBS(−) supplemented with 0.25% trypsin and 50 U ml^−1^ collagenase I at 37 °C for 10 min, followed by dissociation into single cells by gentle pipetting. Bioengineered salivary glands were reconstituted using submandibular gland and sublingual gland cells according to the organ germ method previously reported^26^. Briefly, the bioengineered salivary glands were placed onto a cell culture insert (0.4 μm pore diameter; BD) and incubated at 37 °C for 3 days in DMEM/F-12 (1:1 mixture of DMEM and Ham’s F-12; KOJIN BIO, Saitama, Japan) containing 10% fetal bovine serum (GIBCO, Grand Island, NY, USA), 100 U ml^−1^ penicillin (Sigma-Aldrich) and 100 μg ml^−1^ streptomycin (Sigma-Aldrich). To form interepithelial tissue connections between the host parotid duct and the bioengineered salivary gland, a polyglycolic acid (PGA) monofilament thread guide (9–0 PGA absorbable surgical suture; Gunze, Kyoto, Japan) was appended into a bioengineered salivary gland germ by insertion into the epithelial and mesenchymal portions.

### Transplantation

To prepare salivary gland defect mice, the parotid, submandibular and sublingual glands of 7-week-old C57BL/6 female mice were extracted under deep anaesthesia. Next, the bioengineered salivary gland germ containing a PGA monofilament guide was arranged on the masseter muscle. To create connections between the host parotid duct and the epithelial tissue of the bioengineered salivary gland, our previously developed interepithelial tissue connection method was modified. Briefly, the PGA monofilament guide was inserted into the host parotid duct, and collagen gel and masseter muscles were fixed using nylon thread (8–0 black nylon 4 mm 1/2R, Bear Medic Corp., Ibaraki, Japan).

### Analysis of duct connection using FITC-gelatine conjugate

Fluorescein 5-isothiocyanate (FITC; Dojindo, Kumamoto, Japan) was conjugated to gelatine (Sigma-Aldrich). Briefly, 10 mg of FITC was dissolved in 1 ml dimethylsulphoxide (Sigma-Aldrich) at pH 11. FITC solution and 20% (w/v) gelatine solution at pH 11 were mixed for conjugation at 37 °C overnight. The unconjugated FITC was then removed using a NAP-25 column (GE Healthcare UK Ltd., Buckinghamshire, England). Then, 8 μλ of filtered FITC-conjugated gelatine was injected into the host parotid duct using a FemtoJet microinjector (Eppendorf, Tokyo, Japan) under a stereo Lumar V12 and AxioCam fluorescent stereoscopic microscope system (Carl Zeiss, Oberkochen, Germany).

### Histochemical analysis and immunohistochemistry

The tissues were removed and immersed in Mildform 10N (Wako, Osaka, Japan), processed for standard paraffin embedding and sectioned at 5 μm. Tissue sections were stained with haematoxylin–eosin or periodic acid–Schiff and observed using an Axioimager A1 microscope (Carl Zeiss) and an AxioCAM MRc5 (Carl Zeiss).

For fluorescent immunohistochemistry, frozen sections (10-μm section for AQP/E-cadherin and calponin/E-cadherin double staining, 100-μm section for NF-H/calponin double staining and 200-μm section for duct connection analysis) were prepared and immunostained as previously described^27^. Briefly, the following primary antibodies were used: E-cadherin (1:50, mouse, BD); AQP5 (1:100, rabbit, Millipore, Billerica, MA, USA), calponin (1:250, rabbit, Abcam, Cambridge, MA, USA); α-SMA (1:100, rabbit, Abcam) and NF-H (1:500, rat, Millipore) in blocking solution (PBS (−) containing 1% bovine serum albumin and 0.01% TX-100). The primary antibodies were detected by incubation with highly cross-absorbed Alexa Fluor 594 Goat Anti-Rabbit IgG (H+L; 1:500, Life Technologies, Carlsbad, CA, USA) and Alexa Fluor 488 Goat Anti-Rat IgG (H+L; 1:500, Life Technologies) with Hoechst 33342 dye (1:500, Life Technologies) for 1 h at room temperature. All fluorescence microscopy images were obtained by laser confocal microscopy (LSM780; Carl Zeiss).

### Collection and measurement of saliva secretion

Saliva was collected from the oral cavity using filter paper at 1-min intervals for 25 min after stimulation with intraperitoneal injection of 300 μg kg^−1^ body weight pilocarpine (Wako), and the total amount of saliva was calculated. To perform the amylase western blot assay, the collected saliva was diluted with PBS(−). The samples were immediately stored at −30 °C.

### Analysis of saliva secretion by gustatory stimulation

Gustatory stimulation was conducted using a crystal powder of citrate (Ken-ei Pharmaceutical Co. Ltd., Osaka, Japan). The oral cavity of natural mice or mice engrafted with a bioengineered submandibular gland was wiped off, and then 5 mg of citrate was administered into the oral cavity. After stimulation for 1 min, the secreted saliva was collected from the oral cavity using filter paper for 5 min^29^, and the total amount of saliva was calculated by subtracting the amount of unstimulated saliva secretion.

### Amylase western blot assay

Samples were separated by 10% SDS-PAGE and transferred onto polyvinylidene difluoride membranes using a semidry transblot system (Bio-Rad, Hercules, CA, USA). Western blotting was performed using standard procedures with antibodies against alpha-amylase (1:8,000, Sigma-Aldrich) and peroxidase-conjugated donkey anti-rabbit IgG (1:10,000, Jackson ImmunoResearch Lab, West Grove, PA, USA) diluted in Can Get Signal immunoreaction enhancer solution (TOYOBO, Osaka, Japan). Proteins were detected using the ECL plus Western Blotting Detection Kit (Roche, Basel, Schweiz) as instructed. Images were captured with a Luminescent Image Analyzer LAS-3000 (Fujifilm, Tokyo, Japan). Images were processed and quantified with Multi Gauge software (Fujifilm).

### Amylase activity assay

The amylase activity of secreted saliva was determined using the α-Amylase Assay Kit (Kikkoman Corp., Chiba, Japan) with 2-chloro-4-nitrophenyl 6^5^-azido-6^5^-deoxy-β-maltopentaoside as the substrate according to the manufacturer’s instructions. The production of 2-chloro-4-nitrophenol was detected at 400 nm using a Spectrophotometer GeneQuant 1300 (GE Healthcare Japan, Tokyo, Japan). One unit (U) of α-amylase activity was defined as the release of 1 μmol of 2-chloro-4-nitrophenol within 1 min.

### Visualization of saliva secretion

The oral cavity was washed with water 1 h before analysis. Under deep anaesthesia, fluorescent sodium test paper with a size of 1 × 1.5 mm^2^ (FLUORES Ocular Examination Test Paper 0.7 mg, Showa Yakuhin Kako Co., LTD., Tokyo, Japan) was placed on the tongue surface. After intraperitoneal injection of 300 μg kg^−1^ body weight pilocarpine (0 min), fluorescence microscopy images were captured every 10 min for 80 min using a SteREO Lumar V12 and AxioCam fluorescent stereoscopic microscope system (Carl Zeiss). The fluorescent area of the oral cavity omitting the incisors, lips and bilateral buccal mucosa was quantified during the measurement period using Imaris software (Bitplane, Zurich, Switzerland) (Fig. 4a; top columns and left).

### Histochemical analysis of filiform papillae

In this analysis, salivary gland defect mice were intraperitoneally injected with Lactec D solution (Otsuka Pharmaceutical Factory, Tokushima, Japan) to prevent body weight loss due to swallowing dysfunction. Tissue sections were prepared and examined as described above. The thickness of the epithelium in the filiform papilla was measured as the vertical width of the cornified layer at the centre of the connective tissue papillae, which consists of the interior of the papilla, using AxioVision software (Carl Zeiss; Fig. 4c; top columns and right).

### Analysis of the number of oral bacteria

Bacteria were collected from the buccal mucosa by rubbing with a microbrush (Shofu Inc., Kyoto, Japan), which was then soaked in 100-μL 2 × YT medium. Each sample was plated onto an Luria broth (LB) agar plate and incubated at 37 °C for 12–16 h. The number of oral bacteria was calculated based on the number of bacterial colonies.

### Gravimetric measurement of body weight and survival

The salivary gland defect mice were assigned to two groups. One group was given high-viscosity water at 1 Pa·s, which was prepared with ~1 g of Through King-i (Kissei Pharmaceutical Co., Nagano, Japan) in 100 ml water, and the other group was given only water. Bioengineered salivary gland-engrafted mice and normal mice were given water. The body weight of each mouse was measured at 12-h intervals. The number of days and the survival rate were calculated, and breeding of normal and salivary gland defect mice occurred under normal conditions.

### Statistical analysis

We presented data as means±s.e.m or median±max, min. We used two-tailed Student’s *t*-test to determine *P*-values for statistical significance. Survival analysis was used by Kaplan–Meier methods.

## Author contributions

T. Tsuji, M. Oshima and M. Ogawa designed the research plan; M. Ogawa, A.I., Y.S., K.Y., K.I. and K.N. performed the experiments; M. Ogawa, M. Oshima, M.H., T. Tachikawa and T. Tsuji developed new assay systems and discussed the results; A.I., Y.S., K.Y., I.K. and K.N. analysed the data; and T. Tsuji and M. Ogawa wrote the paper.

## Additional information

**How to cite this article:** Ogawa M. *et al.* Functional salivary gland regeneration by transplantation of a bioengineered organ germ. *Nat. Commun.* 4:2498 doi: 10.1038/ncomms3498 (2013)
